# Physical Image Quality Metrics for the Characterization of X-ray Systems Used in Fluoroscopy-Guided Pediatric Cardiac Interventional Procedures: A Systematic Review

**DOI:** 10.3390/children10111784

**Published:** 2023-11-05

**Authors:** Diego Nocetti, Kathia Villalobos, Kevin Wunderle

**Affiliations:** 1 Departamento de Tecnología Médica, Facultad de Ciencias de la Salud, Universidad de Tarapacá, Arica 1010069, Chile; 2 Department of Radiology, Cleveland Clinic, Cleveland, OH 44195, USA

**Keywords:** image quality, interventional radiology, pediatrics

## Abstract

Pediatric interventional cardiology procedures are essential in diagnosing and treating congenital heart disease in children; however, they raise concerns about potential radiation exposure. Managing radiation doses and assessing image quality in angiographs becomes imperative for safe and effective interventions. This systematic review aims to comprehensively analyze the current understanding of physical image quality metrics relevant for characterizing X-ray systems used in fluoroscopy-guided pediatric cardiac interventional procedures, considering the main factors reported in the literature that influence this outcome. A search in Scopus and Web of Science, using relevant keywords and inclusion/exclusion criteria, yielded 14 relevant articles published between 2000 and 2022. The physical image quality metrics reported were noise, signal-to-noise ratio, contrast, contrast-to-noise ratio, and high-contrast spatial resolution. Various factors influencing image quality were investigated, such as polymethyl methacrylate thickness (often used to simulate water equivalent tissue thickness), operation mode, anti-scatter grid presence, and tube voltage. Objective evaluations using these metrics ensured impartial assessments for main factors affecting image quality, improving the characterization of fluoroscopic X-ray systems, and aiding informed decision making to safeguard pediatric patients during procedures.

## 1. Introduction

Interventional cardiology procedures constitute a substantial source of medical radiation exposure globally, with the potential to subject patients to significant radiation levels [1]. This concern is particularly relevant for pediatric patients with congenital heart disease, as they may necessitate multiple imaging studies, such as cardiac catheterization, which can extend examination durations and elevate radiation exposure [2]. Notably, there has been a noteworthy upsurge in the prevalence of prolonged pediatric cardiac interventions, further increasing the risk of radiation exposure in this population, primarily to the skin [3].

Children are inherently more radiation-sensitive due to factors like higher cell proliferation and percentage of undifferentiated cells, which result in increased stochastic effect risk [2]. Furthermore, their longer lifespan increases the lifetime radiogenic cancer risk [1,4]. Studies reveal 2–3 times higher cancer likelihood from radiation exposure before age 15 as compared to adults [5,6,7]. Hence, optimizing radiation doses and image quality is crucial for pediatric interventional cardiology [3,8,9].

Evaluating the radiation dose rates and image quality of X-ray systems utilized in pediatric interventional procedures is of paramount importance to strike a balance between optimal image quality and appropriate doses for these patients [10]. However, the complexity of these systems, which encompass diverse models, technologies, and operational modes, can pose challenges for cardiologists when comparing and selecting the most suitable options for a given procedure and patient size [2,11]. Thoroughly characterizing X-ray systems used in pediatric interventional procedures using phantoms with tissue-equivalent attenuation characteristics and test objects is essential to optimize procedures, guide the selection of protocols and operation modes, and ensure adequate image quality while minimizing radiation exposure to the lowest reasonably achievable levels [1,2,8,11]. This physical characterization can provide valuable data to improve procedures and facilitate informed decision making when selecting protocols and operation modes [10,11].

Regarding image quality assessment, subjective and objective methods are available. Yet subjectivity can be affected by observer performance, monitor settings, and ambient lighting, causing degradation from other independent and often controllable variables. For unbiased evaluation, software capable of displaying and manipulating DICOM images can provide a means to measure averages and deviations in multiple regions of interest (ROIs). This aids acceptance tests, determining action levels, and setting reference values for consistency checks [9].

Within this framework, the image quality metrics commonly considered are signal-to-noise ratio (*SNR*) [1,5,12,13,14], contrast-to-noise ratio (*CNR*) [9,10,11,14] and high-contrast spatial resolution (*HCSR*) [6,8,11,12]. These metrics can help to optimize the settings of X-ray systems for different patient sizes, ensuring comparable image quality while minimizing patient radiation doses [11]. By utilizing these objective measures, healthcare professionals can make informed decisions regarding the selection of protocols and operation modes for pediatric interventional procedures, prioritizing patient safety, and achieving optimal imaging outcomes.

This systematic review aims to provide a comprehensive analysis of the current understanding of physical image quality metrics relevant to X-ray systems in pediatric interventional cardiology, considering the main factors reported in the literature that influence this outcome. The review addresses the following research questions (RQs):

▪ RQ 1: What are the primary physical image quality metrics commonly utilized for characterizing X-ray systems employed in fluoroscopy-guided pediatric interventional cardiac procedures, and what are the prevalent methods employed to measure these metrics?
▪ RQ 2: What factors have been examined in the literature for their impact on physical image quality metrics in characterizing X-ray systems used in fluoroscopy-guided pediatric interventional cardiac procedures?

By thoroughly analyzing the responses to these research questions, this study seeks to clarify the current understanding of physical image quality metrics applicable to X-ray systems used in pediatric interventional cardiology. The findings from this review provide significant insights that can enhance clinical practice and inform future research in this field.

## 2. Materials and Methods

Between March and April 2023, we conducted a systematic literature review, utilizing Scopus and Web of Science as the main sources of information. The search strategy incorporated relevant descriptors, including image quality, signal-to-noise ratio, contrast-to-noise ratio, spatial resolution, pediatric, interventional, and cardiology. The search filters were narrowed to primary articles in English and Spanish, with publication dates ranging from 2000 to 2022.

**Figure 1:**
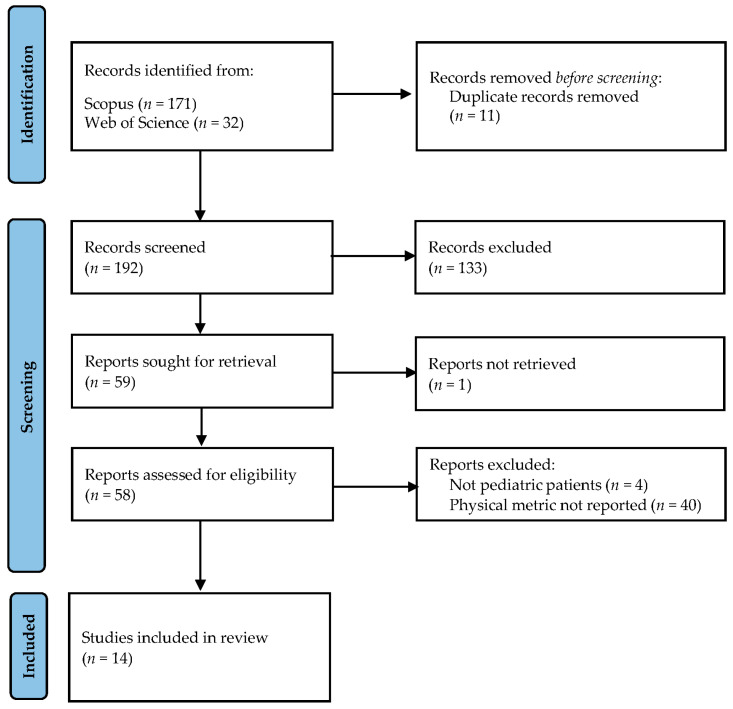
Flowchart of the article selection process in the current systematic review following the PRISMA initiative.

To be included in the review, articles had to meet specific criteria: they (i) evaluated image quality using physical metrics, (ii) focused on characterizing X-ray systems employed in fluoroscopy-guided cardiac interventional procedures, and (iii) involved pediatric patients. Exclusion criteria comprised irrelevant topics, descriptive articles, literature reviews, studies conducted using other imaging techniques or involving adult patients, those lacking objective evaluation of image quality, and those inaccessible in full text. The article selection process followed the guidelines outlined by the PRISMA initiative [15], and it was performed as depicted in Figure 1.

After eliminating duplicate entries, authors D.N. and K.V. conducted a comprehensive evaluation of titles and abstracts. Through collaborative discussions, both authors collectively reached conclusions. When a consensus was established, the articles proceeded to the next stage. If not, a more in-depth assessment of the work’s relevance was conducted before arriving at a final decision.

Once the two main reviewers had agreed on the articles to be included, they independently delved into the full texts. The information was systematically organized, considering the specific metric used to evaluate physical image quality, the assessment methods employed, the angiographic system utilized, factors influencing image quality, and the primary findings of each study. Subsequently, both authors compared their individual decisions regarding the selected articles. Any differences in opinion were thoroughly addressed through further dialogue. Ultimately, all three authors collaboratively determined the final inclusion, resulting in a careful selection of 14 articles for the final analysis.

Regarding the critical appraisal of the included studies, it is important to note that we did not find published tools for experimental studies. However, we deemed it appropriate to develop a specific instrument for this study, consisting of six criteria that addressed aspects of methodology, results reporting, and analysis of potential biases by the authors. Our tool was based on criteria derived from the ARRIVE guidelines 2.0 [16], as well as the Joanna Briggs Institute’s (JBI) Critical Appraisal Checklist for Randomized Controlled Trials [17] and Quasi-Experimental Studies [18]. Specifically, authors D.N. and K.V. independently conducted a manual assessment of bias for each study. Afterward, an overall risk-of-bias rating was assigned to each study. Studies meeting five or six of the evaluated criteria were categorized as having low risk of bias. Those fulfilling three or four criteria were classified as having moderate risk, whereas high risk of bias was linked to works satisfying two or fewer of the analyzed aspects.

## 3. Results

We conducted a systematic review of physical image quality metrics for X-ray systems used in fluoroscopy-guided pediatric interventional procedures. After screening, 14 studies (published between 2000 and 2022) met the inclusion criteria.

### 3.1. Physical Image Quality Assessment

Table 1 displays the selected studies obtained from the search strategy. The table provides essential details, including the last name of the first author, physical image quality metric assessed, test object used, software used for objective evaluation, number of frames analyzed, matrix size, and bit depth. The most frequently reported metric was *SNR* (*n* = 10), followed by *HCSR* (*n* = 8). Additionally, *C* (*n* = 3) and *CNR* (*n* = 3) were also reported, while *N* and SdNR appeared in a smaller number of studies (*n* = 1 each).

### 3.2. Equations Utilized for Physical Image Quality Metrics Calculation

In this section, we present the equations utilized for calculating the physical image quality metrics.

#### 3.2.1. *N* Objective Estimation

The objective estimation of *N* has been reported using Equation (1) [6]:

(1) $$N=\frac{SD_{Bg}}{Bg}$$

where *SD_Bg_* and *Bg* are, respectively, the standard deviation and mean value of the pixels within a ROI located in the proximity of the target structure (e.g., the first low-contrast disk of the test object).

#### 3.2.2. *SNR* Objective Estimation

The most used equation for calculating *SNR* in pediatric interventional cardiology equipment [1,2,5,8,11,13,19,20] is as follows:

(2) $$SNR=\frac{Bg-ROI}{\sqrt{\frac{SD_{Bg}^{2}+SD_{ROI}^{2}}{2}}}$$

where *ROI* and *Bg* represent the average pixel values within a *ROI* located over a structure with higher attenuation than the surrounding structures (background) and in the background, respectively. *SD_ROI_* and *SD_Bg_* denote the standard deviation of pixel values within the same *ROI*. Complementarily, as shown in Equation (3), the study by Bor et al. utilized a formula relying solely on the averages of both the region of interest and background [12].

(3) $$SNR=\frac{ROI-Bg}{\sqrt{ROI+Bg}}$$

Finally, one parameter derived from *SNR* is SdNR, for which the formula retains the numerator of Equation (2) but replaces the denominator with *SD_Bg_* [6].

#### 3.2.3. *C* Objective Estimation

The formula used to determine *C* is as follows [10,11]:

(4) $$C=\frac{Bg-ROI}{Bg}$$

where *ROI* and *Bg* represent the average pixel values within an object of interest with higher attenuation than the surrounding structures (background) and the background itself, respectively. Alternatively, one of the evaluated articles employed the same structure but interchanged the order of subtraction in the numerator of the fraction [12].

#### 3.2.4. *CNR* Objective Estimation

The three articles reporting this metric in the context of pediatric interventional cardiology used different formulas, which are presented below:

(5) $$CNR=\frac{ROI-Bg}{SD_{Bg}}$$

In Equation (5), extracted from article [9], *ROI* represents the average pixel value within an object of interest that has higher attenuation than the background, while *Bg* corresponds to the mean value of the background itself. *SD_Bg_* denotes the standard deviation of the pixels belonging to the background. In contrast, the article by Vañó et al. proposed a formula based on the mean values of the object of interest (*ROI*) and background (*Bg*) [11], resulting in Equation (6):

(6) $$CNR=\frac{Bg-ROI}{Bg^{2}}$$

Finally, in article [10], *CNR* was evaluated using the formula reported for *SNR* in the literature, as shown in Equation (2).

#### 3.2.5. *HCSR* Objective Estimation

The equation utilized in the literature to calculate *HCSR* [1,4,6,8,11,12,13,20] is provided below:

(7) $$HCSR=SD_{1}-SD_{2}$$

where *SD*_1_ denotes the standard deviation of the pixel value within a region inside the bar pattern, typically the seventh group of bars within the TOR 18FG, while *SD*_2_ corresponds to the background within the region of higher attenuation on the bar pattern.

### 3.3. Factors Influencing Image Quality

Table 2 provides an overview of the factors and categories investigated by each selected article obtained from the employed search strategy.

### 3.4. Assessment of Bias Risk

**Figure 2:**
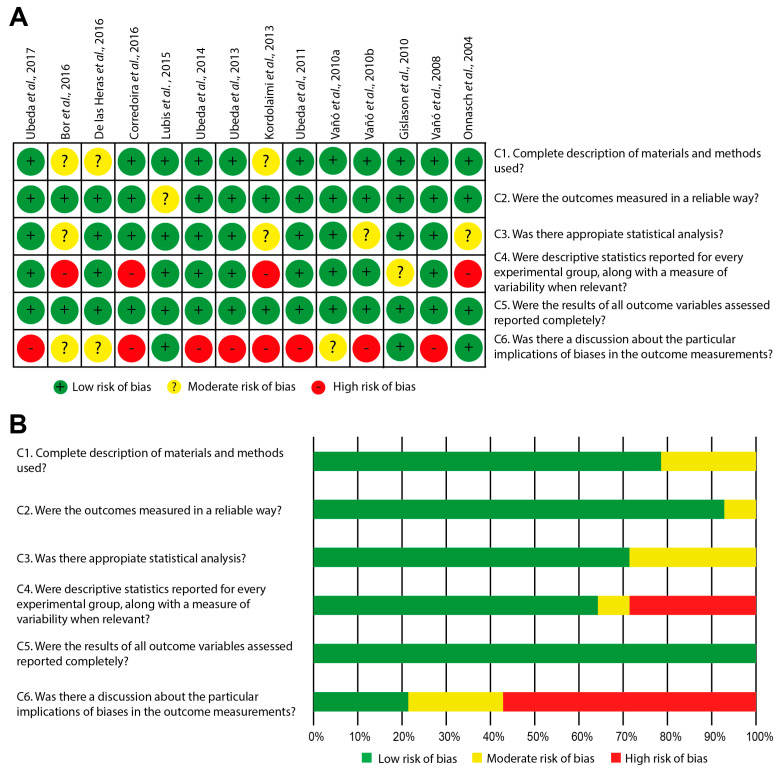
(**A**) Individual risk-of-bias assessment for the articles included in the systematic review (Ubeda et al., 2017 [1]; Bor et al., 2016 [12]; De las Heras et al., 2016 [9]; Corredoira et al., 2016 [13]; Lubis et al., 2015 [5]; Ubeda et al., 2014 [2]; Ubeda et al., 2013 [4]; Kordolaimi et al., 2013 [19]; Ubeda et al., 2011 [8]; Vañó et al., 2010a [20]; Vañó et al., 2010b [6]; Gislason et al., 2010 [10]; Vañó et al., 2008 [11]; Vañó et al., 2004 [7]). (**B**) Summary graph depicting the results obtained for each evaluated criterion.

Figure 2A shows an individual critical appraisal of the selected articles, based on the estimated risk of bias according to six criteria (C1 to C6). Meanwhile, the summary of information for each criterion is presented in Figure 2B. A predominance of studies with low risk of bias was observed for the three criteria evaluating the methodology used (C1–C3), with performance ranging from 71.4% to 92.9%. Among these, C1 revealed a predominance of comprehensive reporting of the materials and methods employed (78.6%), with a low proportion of studies omitting certain details. Although these details hold significance when comparing the results with those of other authors, they do not intrinsically compromise the validity of the results obtained. Criterion C2 exhibited the highest degree of compliance, indicating the use of valid test objects and computational analysis strategies that support the objectivity of the methodology for analyzing physical image quality metrics. Finally, C3 indicated a prevalence of studies carrying out statistical analyses appropriate to the purpose of the work (71.4%), followed by articles that, although they used descriptive statistics, omitted the necessary comparative measures to support the differences between the groups studied.

Conversely, regarding the reporting of measures of outcome variability (C4), it was found that 64.3% of studies met this aspect. Concerning the completeness of reporting outcome variables (C5), it was established that all works successfully fulfilled this criterion. Lastly, the aspect showing the highest bias risk was the discussion of implications related to potential study biases, an area that was scarcely addressed by the analyzed studies (21.4%), with a significant percentage of works omitting this aspect (57.1%), as depicted in Figure 2A,B.

## 4. Discussion

Characterizing angiographic equipment for pediatric use is of utmost importance, given the intricacy of X-ray systems in pediatric interventional procedures [2]. This characterization entails assessing dose and image quality using diverse techniques, such as entrance surface air kerma (K_a,e_) measurement and image quality assessment with test objects. Such evaluations enable cardiologists to identify optimal protocols and operation modes that strike a balance between image quality and dose, thereby minimizing potential long-term tissue damage in vulnerable pediatric patients [1,10]. Additionally, this evaluation facilitates the selection of appropriate operation modes for different procedures and patient sizes, ensuring the safe and effective use of fluoroscopy-guided pediatric cardiac interventions [11].

This article presents a comprehensive analysis of the current understanding of physical image quality metrics relevant to X-ray systems in pediatric interventional cardiology. The information gathered for this purpose is provided below, aligning with the research questions addressed in this study.

### 4.1. What Are the Primary Physical Image Quality Metrics Commonly Utilized for Characterizing X-ray Systems Employed in Fluoroscopy-Guided Pediatric Interventional Cardiac Procedures, and What Are the Prevalent Methods Employed to Measure These Metrics?

Table 1 presents the most evaluated physical image quality metrics in the included studies, comprising *N*, *SNR*, SdNR, *C*, *CNR*, and *HCSR*. These metrics hold significant importance in characterizing the fluoroscopic systems used in pediatric interventional cardiology procedures and are detailed below:

#### 4.1.1. *N*

In digital imaging techniques, brightness variations are attributed to statistical fluctuations in X-ray detection and the electronic chains of digital image receptors, rather than changes in radiation attenuation [21,22]. *N* in fluoroscopy-guided pediatric interventional procedures poses a challenge in distinguishing structures and accurately determining their size [23]. Image degradation in fluoroscopic and cineangiography imaging is mainly caused by two factors: quantum noise, resulting from scattered photons interacting with objects in the X-ray beam, and electronic noise, which originates solely from the detector and remains constant regardless of radiation dose [24].

Out of the assessed articles, only a single study, conducted by Vañó et al. (2010), reported this specific metric [6]. Their conceptual framework was adapted from the work published by Huda et al. (2003), focusing on digital mammography systems [25]. According to Equation (1) [6], lower standard deviation of pixel mean within the ROI corresponds to reduced image noise [23], leading to higher image quality.

#### 4.1.2. *SNR*

Signal refers to the representation of the object of interest (e.g., an artery or stent) depicted in the image [26]. *SNR* serves as an integrative parameter directly related to image quality, encompassing essential aspects such as contrast, spatial resolution, and noise, all contributing to overall image quality [27]. *SNR* plays a crucial role in evaluating images during pediatric interventional cardiology procedures, considering the influence of noise on image quality while quantifying the amount of signal relative to the surrounding tissue [11].

The signal is ideally directly related to the number of photons detected by the image receptor [28]. However, relying solely on the signal is insufficient for a comprehensive image quality assessment. Therefore, *SNR* is commonly employed to evaluate image quality in pediatric interventional cardiology procedures, as it considers the impact of noise on the image while accounting for statistical quantum fluctuations [11]. This parameter exhibits a direct correlation with the square root of the X-ray image receptor dose, allowing for detection of changes in image quality. Notably, alterations in tube voltage or beam filtration influence the distribution of grey levels in an image [7,11].

Most articles that addressed *SNR* calculated it using Equation (2). The authors conducted *SNR* measurements on the TOR 18FG test object [29], comparing the brightness levels of an ROI situated in the first low-contrast disk and another in its vicinity. Onnasch et al. proposed a variant of this measurement by utilizing two distinct measures of *SNR*, namely SNRd and SNRb, which involved calculations in regions with high and low attenuation, respectively. Both SNRd and SNRb remained unaffected by linear grey level windowing, thus demonstrating their robustness as descriptors for image generation and detection quality [7]. However, it is worth noting that the single measurement approach predominated within the analyzed articles.

#### 4.1.3. *C*

This metric represents the difference in signal (brightness level) between a structure of interest and its surroundings. In radiology, it is a relevant parameter because it is directly affected by the selection of the tube voltage (kV) and added filtration used in irradiation [30].

As shown in Table 1, the three articles that evaluated this metric used different test objects. However, based on Equation (4), it is evident that the logic employed to position the ROI was consistent, reflecting through this metric the ability to distinguish two structures that generate a similar level of X-ray attenuation [10,11,12].

#### 4.1.4. *CNR*

This physical parameter assesses an imaging mode’s capability to differentiate between various contrasts present in an acquired image and the inherent noise within that image. A higher *CNR* value indicates a greater ability to distinguish objects such as guide wires or stents within blood vessels. Calculating *CNR* enables an objective evaluation of the visibility of these vessels against the background’s quantum noise [9,11].

The three articles addressing *CNR* utilized different test objects and formulas, as seen in Equations (5) and (6). This lack of consensus regarding the metric is noteworthy, despite the recognized importance of *CNR* in providing valuable insight into image quality and the ability to detect crucial details in the context of medical imaging, which is of outmost importance in the context of pediatric interventional cardiology [9,11].

#### 4.1.5. *HCSR*

Spatial resolution is the ability of an imaging system to represent two adjacent structures as independent elements, involving the recognition of the edge of the structures depicted in the image [22]. In digital systems, this parameter is inversely proportional to the size of the pixels in the array [31]. In techniques such as interventional radiology, a special form of spatial resolution is employed, which refers to the ability to recognize adjacent structures when they exhibit a high difference in attenuating capacity (such as a catheter inside a blood vessel). This is known as *HCSR* [20]. Additionally, in the context of optimizing radiographic procedures, it has also been used to assess differentiation between bone tissue and soft tissue [26] and the ability to visualize small blood vessels [4].

According to Table 1, all the articles included in this review evaluated *HCSR* using a test object consisting of a bar pattern composed of groups of radiolucent and radiopaque bars grouped according to their spatial frequency (0.5–5.0 LP/mm) [1,4,6,8,11,12,13,20]. In general, three frames were evaluated per run, which helps reduce bias associated with the automatic exposure control (AEC) adjustment period in the initial frames [1]. Regarding the formula used to calculate *HCSR* (Equation (7)), there was complete consensus among the published articles.

Finally, another crucial parameter in this context, not addressed by the studies included in the present systematic review, is the Modulation Transfer Function (MTF). MTF is an alternative method for evaluating resolution, closely linked to detector properties, and has been reported in similar contexts within medical imaging [32], thus warranting consideration in this domain.

### 4.2. What Factors Have Been Examined in the Literature for Their Impact on Physical Image Quality Metrics in Characterizing X-ray Systems Used in Fluoroscopy-Guided Pediatric Interventional Cardiac Procedures?

#### 4.2.1. Polymethyl Methacrylate (PMMA) Thickness

PMMA is a material often used to simulate water equivalent tissue thickness. Within this systematic review, the focus lay on the examination of PMMA thickness as a factor influencing image quality, particularly in relation to *SNR* (Table 1 and Table 2). Several studies analyzed the correlation between *SNR* and the thickness of irradiated PMMA. Vañó et al. characterized a pediatric biplane angiographic system with image intensifier (II) technology and observed a trend of decreasing *SNR* with increasing PMMA thickness (4, 8, 12, 16, and 20 cm) [11]. Similarly, three studies by Ubeda et al. yielded analogous trends when evaluating different devices [8]. Corredoira et al. evaluated the impact of PMMA thickness in a biplane angiographic system with flat panel detector (FPD) technology for pediatric interventional cardiology and reported *SNR* behavior consistent with previous findings [13]. Lubis et al. reported similar trends [5].

Furthermore, the investigation by Gislason et al. explored *C* in the cardiac catheterization laboratory, utilizing tin as a substitute for iodine-based contrast in an FPD system. The study involved PMMA thicknesses of 8.5, 12, and 16 cm to approximate typical pediatric patient chest sizes [10]. Vañó et al. examined the impact of increasing PMMA thicknesses (ranging from 4 to 20 cm in 4 cm intervals) on contrast in a biplane angiograph equipped with an II system. The contrast showed a direct relationship with dose increase between each operating mode, except for one of them, where a fitting error was detected through system characterization [11].

All studies reporting *HCSR* analyzed the influence of PMMA thickness on this metric [1,4,6,8,11,12,13,20]. The results consistently indicated that as the PMMA thickness increased, the *HCSR* tended to decrease, primarily due to the influence of scatter radiation [13].

Additionally, De las Heras et al. aimed to objectively evaluate image quality in digital subtraction angiography (DSA) using the International Electrotechnical Commission (IEC) type B phantom [33] by measuring *CNR*. They introduced two scores for overall image quality. CNR_dif_ measured the difference between maximum and minimum *CNR* values, assessing the X-ray system’s ability to distinguish tissues with similar absorption properties. CNR_sum_ combined four *CNR* values, reflecting vessel visibility. Key findings showed low uncertainties for both metrics, facilitating the relationship between examination of physical and clinical image quality [8].

#### 4.2.2. Operation Mode

Operational modes are programs provided with interventional cardiology equipment, defining various aspects of irradiation, and are determined by each manufacturer. Typically, interventional cardiology systems offer three fluoroscopy operational modes: low (FL), medium (FM), and high (FH) dose, along with a cine mode (CI) [5]. Properly adjusting the system in terms of dose is expected to lead to an improvement in image quality as one moves from one operational mode to the next [1]. According to Table 2, this factor represents the second most frequently reported variable, and the studies consistently demonstrated similar results, indicating a reduction in image quality in terms of *HCSR*. This reduction has been attributed to the increase in dose per frame, leading to a consequent reduction in quantum noise [1,4,11].

While the configuration of each mode may vary among systems, evidence suggests that evaluating image quality along with measuring dose levels can alert one to system misalignments or malfunctions. For instance, Vañó et al. reported an inconsistency in the dose and image quality changes for the FM mode compared to the other operational modes, highlighting the need for adjustment in this mode [11].

#### 4.2.3. Anti-Scatter Grid

The study conducted by Ubeda et al. analyzed the impact of using an anti-scatter grid on image quality in a biplane system based on II technology, considering four PMMA thicknesses (4, 8, 12, and 16 cm). The use of the anti-scatter grid resulted in an average 14% increase in *SNR*, and it also revealed that changes in *HCSR* are more noticeable with grid utilization, particularly in CI mode [4]. Similarly, the article by Bor et al. evaluated grid performance parameters in *C*, *SNR*, and *HCSR* for digital fluoroscopic examinations. Based on the obtained results, it was suggested that *C* and *HCSR* were better at highlighting differences between the evaluated types of grids than *SNR* [12].

The 2004 study by Onnasch et al. recommended the use of grids for all patients, including infants [7]. However, subsequent research suggested the need to use grids selectively, based on patient thickness and acquisition protocols [4], additionally, these studies emphasized the significance of adjusting image contrast to remove grids in cases of critical radiation dose [12].

#### 4.2.4. Tube Voltage

Technical parameters of the examination, such as tube voltage, influence the intensity and energy of photons reaching the image receptor and, consequently, impact *SNR* values [8]. In that sense, SNRb and SNRd exhibit different dependencies on X-ray voltage. SNRb shows higher values at low voltages but they decrease rapidly with rising voltage. On the other hand, SNRd reaches its maximum at approximately 79 kV. Based on these metrics, specific voltage ranges are recommended for use in pediatric interventional cardiology procedures [7].

An inverse relationship between *CNR* values and tube voltage was observed, with *CNR* decreasing as tube voltage increased [10]. A similar trend was noted for *HCSR* in PMMA blocks thicker than 5 cm, although the rate of decrease became less pronounced with increasing thickness. The utilization of a grid in conjunction with 5 cm thickness resulted in an increase in *HCSR* with higher tube voltages for all grids, and *HCSR* of images obtained without grids showed a slight improvement [12].

#### 4.2.5. Field of View (FOV)

Regarding the FOV size, it had a significant impact on signal capture. The combination of 48 cm and 42 cm FOVs in fluoroscopy mode improved *SNR* but reduced spatial resolution. Conversely, utilizing 32 cm and 22 cm FOVs with unbinned pixels maintained spatial resolution at the cost of lower *SNR*. Notably, the most substantial *SNR* difference between fluoroscopy and CI modes was observed with 32 cm FOV, resulting in lower fluoroscopy *SNR* overall [13].

In terms of *HCSR*, this article also indicated that *HCSR* increased as FOV decreased due to magnification using unbinned pixels. In CI mode, there was no observed increase in resolution as magnification was electronically performed from 48 to 32 cm FOV. For 22 cm FOV and pediatric protocols, fluoroscopy mode provided higher resolution than CI mode, attributed to the differences in focal spot size according to the mode utilized [13]. In a similar vein, electronic magnification by changing the FOV from 22 to 16 cm significantly improved *HCSR* but also increased the entrance dose by a factor of 1.9 for CI mode in the evaluated system [11].

#### 4.2.6. Vessel Diameter

Iodine contrast was used to assess *SNR* in vessels of different diameters. Generally, high *SNR* was achieved in all vessel sizes when imaged in HD mode with any iodine concentration. HD mode was recommended for superior image quality, but LD mode provided adequate image quality and may be suitable for routine use. Measuring *SNR* in 1 mm vessels was particularly challenging, but an increasing trend in detectability was observed. Hence, further analysis in small arterial vessels is warranted due to the vessel size in young patients [5].

#### 4.2.7. Frames Per Second

Despite the reported increase in dose when modifying the frames per second from 15 to 30, minimal differences in *SNR* were observed in the obtained images. It is possible that another metric could better reflect the change in incident photons on the detector; however, caution must be exercised when interpreting this result as the article did not provide a statistical comparison between both conditions [5].

#### 4.2.8. Contrast Mode

The evaluated system had a post-acquisition filtration algorithm (referred to as ‘low contrast’ and ‘high contrast’ modes) for CI mode, which did not affect the radiological parameters and, therefore, the radiation dose. The evaluation showed a trend towards increased *SNR* values in ‘high contrast’ mode. Nevertheless, similar to the previous point, the information obtained should be approached with caution as no statistical comparison was provided for this variable [5].

#### 4.2.9. Fluoroscopic System

The study conducted by Vañó et al. (2010) revealed that noise values were 40% lower in II-based systems compared to those using FPD systems across all evaluated thicknesses (4, 8, 12, and 16 cm). In terms of efficiency, FPD systems require approximately twice the dose of II-based systems to achieve the same *SNR* value. The FPD-based system exhibited superior performance in *HCSR* compared to II systems, showing higher *HCSR* values ranging from 6.7% to 21.3% for PMMA thicknesses of 8, 12, and 16 cm. These results were attributed to differences between the technologies employed, with II systems utilizing charge-coupled devices (CCD) and FPD systems employing large arrays of amorphous silicon photodiodes and thin film transistors with thallium-doped cesium iodide CsI (Tl) scintillators [6].

During the last decade, the outdated technology of II-based systems has been widely replaced by those based on FPD for fluoroscopic systems intended to be used in interventional procedures. Accordingly, it has been proposed to enhance dose optimization during the transition from II to FPD systems [34]. In general, both types of systems offer advantages for pediatric interventional cardiology, encompassing enhanced ergonomics, patient access, image quality, as well as potential for reduced patient radiation dose [6,19].

#### 4.2.10. Matrix Size

*HCSR* showed a notable decrease in reduced matrices, which could have implications in the clinical interpretation of archived images. This metric demonstrated a robust correlation with visual inspection using magnification on a computer screen, effectively eliminating observer subjectivity. Given the significance of matrix size and pixel pitch in image quality, it is crucial to consider this factor when comparing the impact of other variables reported by studies. In Table 2, a prevalent trend is observed wherein image quality assessments are conducted on smaller-sized matrices (512 × 512), primarily driven by a cost-effective approach to manage extensive image datasets [20].

#### 4.2.11. Filter Thickness

*SNR* can be influenced by the amount of added filtration during irradiation, leading to a slight decrease in *SNR* [24].

Both SNRd and SNRb measurements showed different voltage dependencies. SNRb performed best at low voltages but declined as the voltage increased. By contrast, SNRd decreased at low voltages, resulting in the disappearance of signal difference between Copper (Cu) steps of the test object due to quantum noise at higher amplitudes [7].

#### 4.2.12. AEC Program

In fluoroscopic systems, the AEC system often automatically selects the filtration, tube voltage, and tube charge settings [35]. Proper adjustment of the AEC is crucial for maintaining high image quality, particularly by utilizing the advanced tubes’ high output. Onnasch et al. suggested configuring AEC programs to keep X-ray voltage within an optimal range (above 55 kV but not exceedingly approximately 77 kV) to prevent a decrease in SNRb. Different AEC programs should be tailored for pediatric cardiac angiography to optimize image quality for children of different ages [7].

### 4.3. Final Remarks

One commonly considered parameter in articles on optimization was the Figure of Merit (FOM), which complements objective image quality information with the value of an estimated magnitude in the procedure, such as K_a,e_. Therefore, this parameter aids in assessing the system’s efficiency in terms of the K_a,e_ used to form a radiological image and should be reported together with the evaluated metrics [1,2,5,6,8,10,13,19].

This comprehensive review highlights the importance of optimizing X-ray system configurations and dynamic FPD settings in pediatric cardiac interventional procedures, considering radiation dose levels and specific clinical needs. The utilization of image quality metrics, such as *SNR*, *HCSR*, *C*, and *CNR*, plays a pivotal role in refining imaging parameters and making informed clinical decisions, ultimately leading to improved diagnostic accuracy and enhanced patient care. These metrics not only aid in quality control and technique/system comparisons but also effectively reduce radiation exposure in pediatric patients. Personalized protocols based on image quality metrics can minimize fluoroscopy time while maintaining high image quality. Nevertheless, it is crucial to consider the specific context, as the direct applicability of these metrics may be limited for certain interventional devices. Overall, integrating image quality metrics into medical practice shows significant promise in optimizing pediatric patient dose and image quality, benefiting cardiac X-ray imaging, and advancing medical procedures in this field.

The systematic review’s findings have clinical and practical implications for pediatric healthcare and clinical practice. By understanding the most used physical image quality metrics and their impact on image characteristics, clinicians can optimize imaging protocols and balance image quality and dose for pediatric interventional procedures.

### 4.4. Study Limitations

The most important limitations of this systematic review were as follows. Firstly, the analysis included articles published within a 23-year period, which might have excluded more recent studies with relevant findings. Additionally, preprint studies and unpublished data were not considered, potentially leading to the omission of valuable information. Moreover, the review was limited to studies published in English and Spanish, which could introduce language bias and overlook relevant studies in other languages. The low number of eligible references was another limitation, especially given the broad range of metrics considered, some of which were scarcely reported in the literature.

One of the retrieved articles employed an in-house phantom, created by the researchers themselves, to assess the study’s variables of interest. However, this phantom lacked formal validation, meaning that the results offer trends but do not allow for direct comparisons with other studies. Another potential limitation was that some of the included studies may be dated, which could result in the inclusion of X-ray equipment that is now considered obsolete or rarely used. These systems may not have contemporary relevance in terms of their use. However, it is important to note that this factor was addressed as a fundamental aspect of the development of the concepts analyzed. Furthermore, this consideration is influenced by the parallels that exist between II- and FPD-based technologies.

In addition, some articles omitted important information, such as the program used to extract data from ROIs, number of images analyzed, size of the image matrix, and color depth, which could affect the comprehensiveness of the analysis. Likewise, within the bias analysis (Figure 2), the need to enhance results reporting becomes apparent. This entails incorporating relevant measures of variability and discussing the limitations of studies, thereby facilitating the transparency of aspects that could potentially impact the validity of the obtained results. It is worth noting that the outcomes of studies with high bias risk were only considered when they aligned with findings reported in other studies possessing lower bias risk.

Finally, for some metrics, there was no consensus on the formula to calculate image quality, resulting in different approaches between studies. Consequently, the results may provide trends but require careful interpretation and consideration before applying them to current imaging systems and protocols.

### 4.5. Future Research Directions

The future trends in pediatric interventional cardiology imaging research hold significant potential for enhancing the understanding of the field. Addressing the limitations identified in the systematic review, future studies should consider expanding the inclusion criteria to encompass more research and provide a more comprehensive dataset for analysis. Additionally, researchers are encouraged to validate in-house phantoms before utilization to ensure the accuracy and reliability of results. Furthermore, comprehensive reporting of study details should be considered to improve the transparency and comparability of future investigations. To foster consistency and comparability across studies, the establishment of consensus guidelines or recommendations for calculating and reporting physical image quality metrics is essential. Exploring and developing novel image quality metrics that consider the unique characteristics and imaging needs of pediatric patients could further advance the field. Future researchers should consider examining the relationship between image quality metrics and long-term patient outcomes in pediatric interventional cardiology to shed light on the impact of image optimization on patient care and safety.

## 5. Conclusions

The systematic review provided insight into the use of physical image quality metrics to characterize X-ray equipment in fluoroscopy-guided pediatric interventional cardiac procedures. The evaluation of *N*, *SNR*, *C*, *CNR*, and *HCSR* provided valuable information on the factors influencing image quality and dose in pediatric interventions. By optimizing imaging parameters based on these metrics, clinicians can improve diagnostic outcomes, improve patient safety, and minimize radiation exposure for vulnerable pediatric patients. However, it is important to recognize that these metrics may not fully capture clinical processes, as they do not account for the image visualization process and observer perception of image quality. Nevertheless, they offer a valuable approach to assessing image quality and enrich the quality assessment process, complementing human observer evaluations.

## Figures and Tables

**Table 1:** Selected studies using the search strategy, considering the analyzed physical image quality metrics and other methodological aspects.

First Author (Reference)	Image Quality Metric	Test Object	Software	No. Images Analyzed (Frames)	Matrix Size (Bit Depth)
Ubeda et al. [1]	*SNR, HCSR*	TOR 18FG	Osiris 4.19	3 (10, 12, 15)	512 × 512 (8 bits)
Bor et al. [12]	*SNR, C, HCSR*	LCD4 Hüttner type 18	N.R.	N.R.	N.R.
De las Heras et al. [9]	*CNR*	IEC type B	N.R.	N.R.	N.R.
Corredoira et al. [13]	*SNR, HCSR*	TOR 18FG	ImageJ 1.48r	3 (5, 8, 10)	1024 × 1024 (12 bits)
Lubis et al. [5]	*SNR*	In-house	ImageJ	1 (random)	N.R.
Ubeda et al. [2]	*SNR*	TOR 18FG	Osiris 4.18	3 (10, 12, 15)	1024 × 1024 512 × 512 (8 bits)
Ubeda et al. [4]	*SNR, HCSR*	TOR 18FG	Osiris 4.18	3 (10, 12, 15)	512 × 512 (8 bits)
Kordolaimi et al. [19]	*SNR*	5 mm-thick aluminum plate; TOR 18FG	N.R.	N.R.	N.R.
Ubeda et al. [8]	*SNR, HCSR*	TOR 18FG	Osiris 4.18	3 (10, 12, 15)	512 × 512 (8 bits)
Vañó et al. [20]	*SNR, HCSR*	TOR 18FG	Osiris 4.18	3 (10, 12, 15)	1024 × 1024 (12 bits) 512 × 512 (8 bits)
Vañó et al. [6]	*N*, SdNR, *HCSR*	TOR 18FG	Osiris 4.18	3 (5, 8, 10)	512 × 512 (8 bits)
Gislason et al. [10]	*C, CNR*	Tin detail	Matlab R2008A	40 (1–40)	N.R.
Vañó et al. [11]	*SNR, C, CNR, HCSR*	TOR 18FG	Osiris 4.18	3 (5, 8, 10)	512 × 512 (8 bits)
Onnasch et al. [7]	*SNR*	Patient images	N.R.	1 (1)	512 × 512 (8 bits)

*SNR*, signal-to-noise ratio; *HCSR*, high-contrast spatial resolution; *C*, contrast; *CNR*, contrast-to-noise ratio; *N*, noise; SdNR, signal difference-to-noise ratio. N.R., data not reported.

**Table 2:** Selected studies from the search strategy. Reference, fluoroscopic system, detector system, factors, and categories influencing physical image quality studied by each article.

First Author (Reference)	Fluoroscopic System (Detector)	Factor	Categories
Ubeda et al. [1]	Siemens Artis Zee-Zeego (FPD)	PMMA thickness Operation mode	4, 8, 12, 16, 20 cm FL, FM, FH, CI
Bor et al. [12]	Prototype (FPD)	PMMA thickness	5, 10, 15, 20, 25 cm
		Anti-scatter grid	With 8:1 and 12:1 covered with carbon fiber, 10:1 and 12:1 covered with aluminum, or without grid
		Tube voltage	70, 90, 120 kV
De las Heras et al. [9]	Philips Allura FD 20/10 (FPD)	PMMA thickness	5.7, 25.7 cm
Corredoira et al. [13]	Siemens Artis Zee VC14 (FPD)	Tube voltage Field of view	4, 8, 12, 16, 20 cm 22, 32, 42, 48 cm
Lubis et al. [5]	Philips Allura Xper FD10 (FPD)	Operation mode Vessel diameter Frames per second Contrast mode	FL, FM, FH, CI 0.1, 0.2, 0.4, 0.6, 0.8 cm 15, 30 fps Low, high
Ubeda et al. [2]	Siemens Axiom Artis BC (II)	PMMA thickness Operation mode	4, 8, 12, 16, 20 cm FL, FM, FH, CI
Ubeda et al. [4]	Siemens Axiom Artis BC (II)	PMMA thickness Operation mode Anti-scatter grid	4, 8, 12, 16 cm FL, FM, FH, CI With or without
Kordolaimi et al. [19]	Innova 2100 IQ (FPD) Advantx e E/LC þ DLX (II)	PMMA thickness Field of view	5, 10, 15, 20 cm 12, 15, 17, 20 cm
Ubeda et al. [8]	Siemens Axiom Artis dBC (FPD) Philips Allura Xper FD20 (FPD) Toshiba Rebuilt (II) Siemens Axiom Artis BC (II) General Electric Advantx (II)	PMMA thickness Operation mode	4, 8, 12, 16 cm FL, FM, FH, CI
Vañó et al. [20]	Siemens Axiom Artis dBC (FPD) Siemens Axiom Artis FC (II)	PMMA thickness Matrix size	16, 20, 24, 28 cm 512 × 512, 1024 × 1024
Vañó et al. [6]	Siemens Axiom Artis dBC (FPD) Siemens Axiom Artis BC (II)	PMMA thickness Fluoroscopic system	8, 12, 16 cm FPD, II
Gislason et al. [10]	Allura FD10 (FPD)	PMMA thickness Anti-scatter grid Tube voltage	8.5, 12, 16 cm With or without 50, 55, 60, 65, 70 kV
Vañó et al. [11]	Siemens Axiom Artis BC (II)	PMMA thickness Operation mode Field of view	4, 8, 12, 16, 20 cm FL, FM, FH, CI 16, 22
Onnasch et al. [7]	Philips Integris BH 5000 (II)	PMMA thickness Anti-scatter grid Tube voltage Filter thickness AEC program	8, 11, 15.5, 18.5 cm With or without 50–90 kV 0.2, 0.4 mmCu Program (P) from 1 to 6

FPD, flat panel detector; II, image intensifier; PMMA, polymethyl methacrylate; AEC, automatic exposure control; FL, low fluoroscopy dose; MD, medium fluoroscopy dose; HD, high fluoroscopy dose; CI, cine; fps, frames per second.

## Data Availability

No new data were created or analyzed in this study. Data sharing is not applicable to this article.
